# The Banff Patellofemoral Instability Instrument 2.0: structural validity and responsiveness as a patient-centred tool for assessing recovery after medial patellofemoral ligament reconstruction

**DOI:** 10.1302/2633-1462.711.BJO-2026-0211.R1

**Published:** 2026-11-16

**Authors:** Irene Nga-Lam Sze, Sarah Kerslake, Laurie A. Hiemstra

**Affiliations:** 1 Banff Sport Medicine Foundation, Banff, Canada; 2 Department of Surgery, University of Calgary, Calgary, Canada

**Keywords:** Patellofemoral instability, Disease-specific patient-reported quality of life, MPFL reconstruction, Patellofemoral Instability, knees, IKDC, medial patellofemoral ligament (MPFL) reconstruction, Knee Injury and Osteoarthritis Outcome Score (KOOS), International Knee Documentation Committee, patient-reported outcome measure (PROM), clinicians, surgical stabilization, root mean square

## Abstract

**Aims:**

The study aim was to evaluate the 23-item Banff Patellofemoral Instability Instrument 2.0 (BPII 2.0) to assess patient-reported recovery following medial patellofemoral ligament (MPFL) reconstruction, including its psychometric properties, sub-scale structure, and cross-sectional responsiveness.

**Methods:**

A total of 731 patients (828 knees) undergoing MPFL reconstruction completed BPII 2.0, and a sub-set of 526 patients also completed International Knee Documentation Committee (IKDC) Subjective Knee Form, Knee Injury and Osteoarthritis Outcome Score (KOOS), and Marx Activity Rating Scale (MARS) preoperatively, and at six, 12, and 24 months postoperatively. Evaluation included structural validity via confirmatory factor analysis (CFA), internal consistency, construct validity, and floor/ceiling effects. Cross-sectional responsiveness was assessed using linear mixed models (LMMs).

**Results:**

CFA supported a five-factor structure and hierarchical second-order model (SRMR < 0.08), supporting both BPII 2.0 sub-scale and total scores. Internal consistency was good-to-excellent (Cronbach’s α: total = 0.93 to 0.97; sub-scales = 0.69 to 0.93). Construct validity was strong. Specifically, BPII 2.0 ‘recreation’, ‘lifestyle’, and ‘social/emotional wellbeing’ sub-scales correlated strongly with KOOS-quality of life (*r* = 0.60 to 0.80), and all BPII 2.0 sub-scales correlated strongly with IKDC (*r* = 0.52 to 0.79). No floor/ceiling effects (< 15%) were observed. LMM demonstrated significant improvement across all BPII 2.0 domains through 12 months (p < 0.001), with the most rapid increases between preoperative baseline and six months postoperatively. While ‘work/school concerns’, ‘lifestyle’, and ‘social/emotional wellbeing’ scores improved significantly through 24 months (p < 0.05), remaining sub-scales reached clinical equivalence between 12 and 24 months. ‘Recreation’ and ‘social/emotional wellbeing’ consistently yielded the lowest scores.

**Conclusion:**

The BPII 2.0 is a reliable, valid, and responsive disease-specific instrument for evaluating recovery after MPFL reconstruction. Its multidimensional structure allows assessment of overall and domain-specific outcomes, supporting its use in clinical practice and research across adolescent and adult populations.

Cite this article: *Bone Jt Open* 2026;7(11):1481–1488.

## Introduction

The Banff Patellofemoral Instability Instrument 2.0 (BPII 2.0) is a disease-specific patient-reported outcome measure (PROM) designed to assess quality of life in individuals experiencing patellofemoral instability. The original BPII was introduced in 2013 and refined in 2016 through factor analysis and item reduction, resulting in the development of the BPII 2.0, a shorter, more specific, and user-friendly instrument.^1,2^ The BPII 2.0 consists of 23 questions across five distinct domains: 1) symptoms; 2) work/school concerns; 3) recreation; 4) lifestyle; and 5) social/emotional wellbeing.

Although the instrument has been translated into 13 languages, with the majority demonstrating robust psychometric properties (Appendix A), existing literature has predominantly relied on total scores, leaving sub-scales largely unexplored, even in the original English version. Evaluating the BPII 2.0 at the sub-scale level may provide additional insight into domain-specific aspects of recovery, allowing clinicians and researchers to better understand the physical, functional, and psychosocial factors influencing outcomes in patients with patellofemoral instability.

While the BPII 2.0 has established reliability (e.g. internal consistency and test-retest reliability),^1-3^ its structural validity has not yet been fully evaluated. The existing confirmatory factor analysis (CFA) evaluated a unidimensional structure within a small cohort (n = 100),^4^ and the proposed multidimensional structure has yet to be validated. The lack of structural validity evidence for the BPII 2.0 sub-scales limits clinical interpretation, and leaves the relationship between BPII 2.0 domains and general knee measures largely unexamined. While general knee outcome measures like the Kujala Anterior Knee Pain Scale,^5^ the Knee Injury and Osteoarthritis Outcome Score (KOOS),^6^ and the International Knee Documentation Committee knee scoring system (IKDC)^7^ assess general knee symptoms and function, they often suffer from ceiling effects and fail to capture the disease-specific impact of patellofemoral instability on quality of life.^8^ Because the BPII 2.0 was developed specifically for this population, validating its sub-scales is essential to identify the clinical changes that generic instruments fail to detect.

To date, psychometric evaluation of the BPII 2.0 sub-scales has been limited to preoperative baseline data from four studies.^9-12^ While these studies established strong psychometric properties, little is known about the validity and responsiveness of the sub-scales in the postoperative period. Specifically, it remains unclear whether certain sub-scales exhibit ceiling effects in later stages of recovery, thereby limiting the instrument’s sensitivity to detect clinically meaningful improvements. Furthermore, while previous research identified ‘recreation’ and ‘social/emotional wellbeing’ as the lowest-scoring domains, it remains unknown whether these results are replicable in English-speaking cohorts. Given that recovery following surgical stabilization varies between patients,^13^ the sub-scales may demonstrate distinct score patterns across various postoperative stages. Understanding these patterns is important for interpreting recovery and identifying persistent domain-specific deficits following surgery. These gaps highlight the need for a comprehensive evaluation of the structural and construct validity, as well as the cross-sectional responsiveness, of the BPII 2.0 sub-scales.

The purpose of this study was to evaluate the structural validity, psychometric properties, and cross-sectional responsiveness of the BPII 2.0 and its sub-scales in a large English-speaking cohort of patients with patellofemoral instability undergoing medial patellofemoral ligament (MPFL) reconstruction. Specifically, this study aimed to assess structural validity using CFA, examine construct validity through correlations with established general knee outcome measures, determine internal consistency, and evaluate score distributions for floor and ceiling effects. In addition, changes in total and sub-scale scores were analyzed across four timepoints over a 24-month period to assess cross-sectional responsiveness.

## Methods

Data were extracted from a prospectively collected clinical database which tracked patient-reported recovery following surgical stabilization for patellofemoral instability. Patients undergoing MPFL reconstruction (with or without concomitant bony procedures) between July 2017 and March 2026 were eligible for inclusion, with data collection spanning from 2019 to 2026. Patients completed a comprehensive set of PROMs via an online platform (Ortech Systems, Canada). The extracted dataset included four standardized tracking intervals: preoperative baseline, and at six, 12, and 24 months postoperatively. This study received ethics approval from the University of Calgary Conjoint Health Research Ethics Board (REB15-0616).

### Patient characteristics

A total of 731 patients (828 knees; 97 bilateral surgical cases) who completed the BPII 2.0 at a minimum of one timepoint were included. This sample yielded 1,617 responses across all timepoints, averaging 2.0 responses per knee out of four possible timepoints. From this cohort, a sub-sample of 526 patients (594 knees; 68 bilateral surgical cases; 1,030 responses) completed additional measures for construct validation. Demographic characteristics were comparable between the full cohort and the validation sub-sample (Table I and Table II).

**Table I.** Available assessments for the full cohort and construct validity sub-sample.

Variable	Full cohort, n		Construct validity sub-sample, n	
	**Patients**	**Knees**	**Patients**	**Knees**
**Overall sample**	731	828	526	594
Preoperative baseline	347	365	169	178
6 mths postoperative	419	450	275	300
12 mths postoperative	410	437	277	301
24 mths postoperative	348	365	234	251

**Table II.** Patient demographic characteristics for the full cohort and construct validity sub-sample.

Patient characteristics	Full cohort	Construct validity sub-sample
Sex (female), n (%)	533/731 (72.9)	395/526 (74.8)
Paediatric (< 18 yrs), n (%)	213/731 (29.1)	150/526 (28.6)
Bilateral symptoms, n (%)	281/731 (38.4)	213/526 (40.4)
Median age at surgery, yrs (IQR)	22.2 (17.5 to 30.1)	22.6 (17.6 to 30.9)
Median BMI, kg/m^2^ (IQR)	24.0 (21.0 to 28.1)	24.0 (21.0 to 28.0)
Median age at first dislocation, yrs (IQR)	14.0 (12.0 to 17.0)	14.0 (12.0 to 16.0)
Left operative knee, n (%)	465/828 (56.2)	327/594 (55.1)
Concomitant bony procedure, n (%)	264/828 (31.9)	180/594 (30.3)

Patient characteristics are reported by number of patients, whereas operative-knee characteristics are reported by number of knees.

### Outcome measures

The primary outcome measure was the BPII 2.0, which evaluates five domains: 1) symptoms; 2) work/school concerns; 3) recreation; 4) lifestyle; and 5) social/emotional wellbeing. For each of the 23 items, patients select a point on a visual analogue scale between two anchors (e.g., ‘severe difficulty’ (0) to ‘no difficulty at all’ (100)), where higher scores indicate better quality of life.

In addition to the primary outcome measure, a representative sub-sample completed other established outcome measures: the knee-specific 42-item KOOS (or 39-item KOOS-Child),^14^ the 18-item IKDC (or 20-item Pedi-IKDC),^7^ and the four-item Marx Activity Rating Scale (MARS).^15^ For these measures, higher values represented better knee function, fewer symptoms, or greater physical activity levels.

### Statistical analysis

Prior to analysis, data were screened for missing values and duplicates. Bilateral knees were both included if they were recorded in separate entries across different years. However, when a single record could be used for different timepoints for the left and right knees, the knee at the earlier postoperative timepoint was selected to prevent duplicate patient entries. All statistical analyses were performed using R (v. 4.4.3, R Foundation for Statistical Computing, Austria).^16^ For all tests, statistical significance defined when p < 0.05.

Structural validity was evaluated across four timepoints using CFA via the LaVan package. A unidimensional (one-factor) model was compared with a correlated five-factor model to validate the individual sub-scales, followed by a second-order model to support the use of a composite score. Following Consensus‐Based Standards for the Selection of Health Measurement Instruments (COSMIN) guidelines,^17^ structural validity was considered sufficient if model fit met at least one of the following thresholds: Comparative Fit Index (CFI) or Tucker-Lewis Index (TLI) > 0.95, root mean square error of approximation (RMSEA) < 0.06, or standardized root mean square residual (SRMR) < 0.08. Internal consistency was evaluated using both Cronbach’s alpha (α) and McDonald’s omega (ω), with values ≥ 0.70 indicating acceptable reliability.

Construct validity of the BPII 2.0 was assessed in a representative sub-sample using Pearson correlation coefficients (*r*) with concurrently administered PROMs. For joint-specific measures (i.e., KOOS, IKDC), correlations were analyzed at the knee-case level. In bilateral cases, the single BPII 2.0 entry was paired separately with each individual knee-specific score.

Floor and ceiling effects were defined as > 15% of patients reaching minimum or maximum scores.^18^ Cross-sectional responsiveness across timepoints was analyzed using a linear mixed model (LMM) via the lme4 package to evaluate score variations from preoperative baseline to 24 months, supplemented by Cohen’s *d* effect sizes.^19^ The LMM was preferred over the standardized response mean (SRM) as it maximized the use of all available data to handle unbalanced data across timepoints and accounted for within-subject correlation. Specifically, a nested random intercept (1 | Patient_ID / Knee_Side) was used to account for repeated measures over time and the clustering of patients who underwent staged bilateral surgeries. Fixed effects included time, sub-scale, and their interaction. Degrees of freedom and p-values were calculated using the Satterthwaite approximation, with post-hoc pairwise comparisons performed using the Tukey honestly significant difference (HSD) method to assess score variations across different timepoints within each domain, and to identify differences between sub-scales at each timepoint.

To assess the potential influence of bilateral cases on structural validity and responsiveness, a sensitivity analysis was performed across all analyses by comparing the original data with a sub-set where a single knee was randomly selected for each bilateral case. The only exception was the construct validity analysis, which retained all knee-level data to correlate with joint-specific measures.

Sample size was determined according to COSMIN.^17^ To achieve an ‘excellent’ rating for structural validity, a 7:1 subject-to-item ratio was required (161 participants for the 23-item BPII 2.0). The study sample consistently exceeded this requirement, as well as the n > 100 threshold for providing stable estimates for internal consistency, construct validity, and floor/ceiling effects.^17^

## Results

### Structural validity

CFA supported the five-factor structure of the BPII 2.0 across all timepoints, consistently meeting acceptable thresholds (SRMR < 0.08). The superior fit of this multidimensional model over the one-factor model justifies the use of sub-scale-specific clinical interpretation. Moreover, the comparable fit of the hierarchical second-order model established the structural validity of a composite score in addition to individual sub-scales (Table III).

**Table III.** Confirmatory factor analysis fit indices for the Banff Patellofemoral Instability Instrument 2.0 from preoperative baseline to 24-month follow-up (n = 1,617).

Time	Models	*X* ^2^	*df*	p-value	CFI	TLI	RMSEA	SRMR
Preoperative baseline (n = 365)	1-factor	1,485.35	230	< 0.001	0.742	0.716	0.122	0.075
	5-factor*	981.22	220	< 0.001	0.843	0.820	0.097	0.067
	Second-order	1,008.85	225	< 0.001	0.839	0.819	0.098	0.069
6 mths (n = 450)	1-factor	2,063.77	230	< 0.001	0.744	0.718	0.133	0.077
	5-factor*	1,102.61	220	< 0.001	0.877	0.858	0.094	0.061
	Second-order	1,118.98	225	< 0.001	0.875	0.859	0.094	0.063
12 mths (n = 437)	1-factor	1,806.15	230	< 0.001	0.811	0.792	0.125	0.062
	5-factor*	1,005.20	220	< 0.001	0.906	0.892	0.090	0.049
	Second-order	1,060.87	225	< 0.001	0.900	0.887	0.092	0.052
24 mths (n = 365)	1-factor	1,521.89	230	< 0.001	0.828	0.810	0.124	0.056
	5-factor*	895.02	220	< 0.001	0.910	0.896	0.092	0.043
	Second-order	928.44	225	< 0.001	0.906	0.894	0.093	0.046

* The five-factor structure was identified as the best-fitting model across all timepoints based on fit indices.

CFI, Comparative Fit Index; *df*, degrees of freedom; RMSEA, root mean square error of approximation; SRMR, standardized root mean square residual; TLI, Tucker-Lewis Index; X^2^, chi-squared.

Standardized factor loadings (λ) ranged from 0.46 to 0.94, with most items showing moderate-to-strong associations with their respective latent constructs (λ > 0.50). Only two items (items 3 and 4 at preoperative baseline) fell below the 0.50 threshold. In addition, factor correlations were below 0.85 at preoperative baseline, suggesting the factors are distinguishable at the preoperative stage. While higher correlations (*r* > 0.85) were observed between the ‘lifestyle’, ‘recreation’, and ‘social/emotional wellbeing’ sub-scales at postoperative timepoints, these high inter-factor correlations are consistent with the expected overlap within a second-order structure and with parallel improvement across domains following surgical stabilization. Standardized factor loadings and factor correlations are summarized in Appendix B.

### Internal consistency

The BPII 2.0 total score and its five sub-scales demonstrated good to excellent internal consistency at both preoperative and postoperative stages (Appendix B). Both Cronbach’s α and McDonald’s ω ranged from 0.93 to 0.97 for the total score and 0.72 to 0.93 for the individual sub-scales. All values exceeded the acceptable threshold of 0.70, confirming the BPII 2.0 total score and its five sub-scales as a reliable tool for tracking recovery at different timepoints up to two years postoperatively in this patient population.

### Construct validity

Using the sub-sample (526 patients, 594 knees), the BPII 2.0 total score demonstrated strong correlations with the KOOS ‘quality of life’ (*r* = 0.66 to 0.82) and IKDC (*r* = 0.74 to 0.84) across all timepoints. Analysis of the BPII 2.0 sub-scales revealed domain-specific associations that establish their construct validity. The BPII 2.0 ‘symptoms’ sub-scale showed its strongest associations with KOOS ‘pain’ and ‘symptoms’ (*r* = 0.59 to 0.79), while the BPII 2.0 ‘recreation’, ‘lifestyle’, and ‘social/emotional wellbeing’ sub-scales demonstrated their strongest relationships with KOOS ‘quality of life’ and ‘sport/recreation function’ (*r* = 0.58 to 0.80), consistent with the sub-scale constructs (Table IV).

**Table IV.** Pearson correlation coefficients (r) between the Banff Patellofemoral Instability Instrument 2.0 (BPII 2.0) and Knee Injury and Osteoarthritis Outcome Score (KOOS), International Knee Documentation Committee knee scoring system (IKDC), and Marx Activity Rating Scale (MARS) at preoperative baseline and follow-up intervals in a sub-sample.

BPII 2.0 item	KOOS (n = 133 to 224)					IKDC (n = 134 to 219)	MARS (n = 174 to 298)
	SYM	Pain	ADL	Sport/ Rec	QoL		
**Total**							
Preoperative baseline	0.53*	0.58*	0.61*	0.65*	0.66*	0.74*	0.06
6 mths	0.65*	0.72*	0.71*	0.72*	0.80*	0.80*	0.13†
12 mths	0.66*	0.74*	0.69*	0.76*	0.79*	0.80*	0.40*
24 mths	0.70*	0.76*	0.75*	0.83*	0.82*	0.84*	0.34*
**Symptoms**							
Preoperative baseline	0.59*	0.59*	0.52*	0.56*	0.49*	0.67*	0.00
6 mths	0.63*	0.68*	0.63*	0.58*	0.56*	0.67*	0.07
12 mths	0.58*	0.64*	0.55*	0.57*	0.60*	0.63*	0.28*
24 mths	0.73*	0.79*	0.77*	0.74*	0.75*	0.79*	0.25*
**Work/school concern**							
Preoperative baseline	0.41*	0.49*	0.52*	0.55*	0.40*	0.60*	-0.05
6 mths	0.41*	0.50*	0.55*	0.49*	0.49*	0.55*	0.12†
12 mths	0.54*	0.66*	0.64*	0.69*	0.60*	0.67*	0.24*
24 mths	0.58*	0.68*	0.71*	0.75*	0.62*	0.72*	0.28*
**Recreation**							
Preoperative baseline	0.35*	0.37*	0.43*	0.52*	0.62*	0.58*	0.13
6 mths	0.55*	0.60*	0.57*	0.65*	0.77*	0.75*	0.14†
12 mths	0.60*	0.62*	0.57*	0.68*	0.76*	0.74*	0.44*
24 mths	0.65*	0.69*	0.67*	0.77*	0.80*	0.78*	0.36*
**Lifestyle**							
Preoperative baseline	0.41*	0.46*	0.53*	0.55*	0.61*	0.62*	0.11
6 mths	0.62*	0.67*	0.66*	0.67*	0.77*	0.74*	0.13†
12 mths	0.60*	0.70*	0.68*	0.70*	0.75*	0.75*	0.38*
24 mths	0.62*	0.69*	0.70*	0.78*	0.75*	0.78*	0.32*
**Social/emotional wellbeing**							
Preoperative baseline	0.41*	0.42*	0.44*	0.45*	0.58*	0.53*	0.07
6 mths	0.57*	0.59*	0.57*	0.61*	0.73*	0.66*	0.06
12 mths	0.63*	0.66*	0.61*	0.72*	0.77*	0.75*	0.38*
24 mths	0.58*	0.61*	0.58*	0.72*	0.76*	0.71*	0.32*

* p < 0.01.

† p < 0.05.

ADL, activities of daily living; QoL, quality of life; Sport/Rec, sport and recreation function; SYM, symptoms.

The BPII 2.0 demonstrated comparable correlation strengths with corresponding paediatric-specific measures (KOOS-Child and Pedi-IKDC), despite the smaller sample sizes available for these instruments (n = 31 to 70). The BPII 2.0 total score showed moderate-to-strong correlations with the KOOS-Child ‘quality of life’ (*r* = 0.62 to 0.75) and the Pedi-IKDC (*r* = 0.41 to 0.77). Similarly, the BPII 2.0 ‘recreation’, ‘lifestyle’, and ‘social/emotional wellbeing’ sub-scales were particularly well-aligned with the KOOS-Child ‘quality of life’ and ‘sport/recreation function’ (*r* = 0.32 to 0.76) (Appendix C).

In contrast to these general knee outcome measures, discriminant validity was supported by the relationship with the MARS. Correlations with the BPII 2.0 total score were non-significant at preoperative baseline (*r* = 0.06) and reached weak-to-moderate statistical significance postoperatively (*r* = 0.13 to 0.40). The BPII 2.0 ‘recreation’ sub-scale demonstrated the strongest relationship with MARS at 12 and 24 months, reflecting the expected association between these two measures as patients return to physical activity. These findings confirm that the BPII 2.0 sub-scales capture clinically meaningful domains of patient-reported recovery.

### Floor/ceiling effects and cross-sectional responsiveness

Using the full cohort (731 patients, 828 knees), the BPII 2.0 total score and sub-scales exhibited no floor or ceiling effects, consistently remaining below the 15% threshold (Appendix D). Cross-sectional responsiveness was then examined to test whether the BPII 2.0 sub-scales are sensitive enough to detect clinical changes and distinct recovery pathways at different postoperative stages.

The LMM revealed a significant time × sub-scale interaction (F_12, 7222.48_ = 11.03, p < 0.001), indicating that recovery trajectories differed significantly across the five BPII 2.0 domains. Significant main effects were also observed for time (F_3, 7900.99_ = 1,144.35, p < 0.001) and sub-scale (F_4, 7222.48_ = 503.99, p < 0.001).

Post-hoc pairwise comparisons were conducted to evaluate the BPII 2.0 sub-scales’ sensitivity to both cross-sectional change and domain-specific differences. Th greatest improvement occurred between preoperative baseline and six months across all domains (all Tukey-adjusted p < 0.001), characterized by moderate-to-large effect sizes (Cohen’s *d* range: 0.71 to 1.29). While significant improvements continued for all sub-scales through 12 months, only the ‘work/school concerns’ sub-scale demonstrated further significant improvement between 12 and 24 months with a small effect size (*d* = 0.20). During the same interval, the ‘lifestyle’ and ‘social/emotional wellbeing’ sub-scales reached statistical significance, but their effect sizes remained negligible (*d* ≤ 0.13). In contrast, the ‘symptoms’ and ‘recreation’ sub-scales reached a plateau, showing similar scores (*d* ≤ 0.06) between the 12- and 24-month intervals.

Pairwise comparisons revealed significant differences between the scores of most BPII 2.0 sub-scales across all timepoints (all Tukey-adjusted p < 0.05). The ‘symptoms’ and ‘work/school concerns’ sub-scales consistently yielded the highest scores. ‘Lifestyle’ sub-scale improved steadily and remained significantly lower than ‘symptoms’ in the early stages of follow-up. Consistent with prior BPII studies, the ‘recreation’ and ‘social/emotional wellbeing’ sub-scales remained significantly lower than all other sub-scales across all timepoints (all Tukey-adjusted p < 0.001).

Sensitivity analysis evaluating a sub-set constrained to only one knee per patient revealed that the results for structural validity, internal consistency, floor/ceiling effects, and responsiveness were consistent with the primary findings.

## Discussion

This study evaluated the structural validity, psychometric performance, and cross-sectional responsiveness of the BPII 2.0 total score and sub-scales in a large English-speaking cohort of patients undergoing MPFL reconstruction. The findings demonstrate that BPII 2.0 possesses strong measurement properties, including confirmation of the proposed five-factor structure, excellent internal consistency, and strong construct validity relative to established general knee outcome measures. Importantly, the instrument demonstrated no floor or ceiling effects, and was responsive to change across all domains over the two-year postoperative period. These results support the use of both the BPII 2.0 total score and five sub-scales to assess patient recovery following MPFL reconstruction, and provide clinically relevant insight into domain-specific recovery.

The BPII 2.0 exhibits robust psychometric properties. CFA validated a stable five-factor structure, providing empirical support for the independent interpretation of both sub-scales and the total score. This structural validity is further strengthened by high internal consistency and strong construct validity with established knee PROMs. Collectively, these results demonstrate that BPII 2.0 is a precise, multidimensional measure that provides clinically meaningful insights into the specific domains of a patient’s recovery and quality of life.

A key finding in this study is that the BPII 2.0 ‘social/emotional wellbeing’ and ‘recreation’ sub-scales consistently yielded the lowest scores, indicating that psychological recovery and sport reintegration may lag behind physical functional recovery. This aligns with existing literature on kinesiophobia and pain catastrophizing, where fear of reinjury persists despite successful surgical stabilization.^20,21^ By identifying these specific deficits through sub-scale analysis, clinicians can guide targeted interventions, such as psychological support or sport-specific physiotherapy for patients struggling in these domains. Furthermore, these findings provide a valuable framework for clinicians to manage patients’ expectations, highlighting that psychological confidence often develops at a different pace than physical recovery.

The BPII 2.0 demonstrates superior sensitivity for patellofemoral instability compared with region-specific measures. While the BPII 2.0 showed no floor or ceiling effects, the study data and existing literature indicate the KOOS/KOOS-Child ‘activity of daily life’ domain frequently reaches a ceiling in this clinical population, reflecting the focus on basic functional tasks.^12^ Specifically, the study data revealed that the adult KOOS reached a ceiling effect by 12 months, while the KOOS-Child reached a ceiling prior to surgery. Conversely, the IKDC lacks domain-specific insights, and its complex scoring system may increase respondent burden. These results suggest that disease-specific PROMs, such as the BPII 2.0, are essential for providing the sensitivity required to capture the high-level functional improvements that region-specific instruments fail to detect in the patellofemoral instability population.

The BPII 2.0 uses a single version for both paediatric and adult patients. Its 8.9 grade reading level, typically corresponding to ages 13 to 15 years, is age-appropriate for this clinical population and reduces the administrative burden of switching between ‘Child’ and ‘Adult’ versions as patients grow.^22^ This ensures that longitudinal data remain directly comparable from adolescence through to adulthood. As a disease-specific instrument, the BPII 2.0 features questions more relevant to patients with patellofemoral instability and significantly reduces respondent burden for those with bilateral conditions. Unlike region-specific measures such as the KOOS and IKDC, which require repetitive questionnaires for each knee, the BPII 2.0 is a useful and valid tool for capturing the global impact of the condition. Furthermore, the study data indicate that distinct recovery patterns across sub-scales, along with domain-specific differences at individual timepoints, may be useful to guide clinical decision-making and tailor postoperative rehabilitation to a patient’s specific needs.

A challenge in orthopaedic research is the need for multiple PROMs to capture the full scope of recovery, including psychological readiness and physical activity levels. While a battery of tools provides a comprehensive evaluation, it increases patient burden and administrative complexity. The results of this study demonstrate that the BPII 2.0 is a highly valid, reliable, and responsive instrument that effectively captures multiple domains of recovery within a single measure. By providing both a total score and sub-scale data, the BPII 2.0 can serve as a standalone patient-reported outcome tool.

This study has several strengths. First, it evaluated the BPII 2.0 and its sub-scales in a large cohort of patients with patellofemoral instability, exceeding recommended sample sizes for psychometric evaluation. Second, structural validity was assessed using CFA across multiple timepoints, providing robust evidence for the multidimensional structure of the BPII 2.0. Third, the inclusion of multiple postoperative timepoints enabled the evaluation of cross-sectional responsiveness over a two-year recovery period, reflecting the typical follow-up timeline after MPFL reconstruction. Finally, the inclusion of both adolescent and adult patients demonstrates the applicability of the BPII 2.0 across a broad age range within the patellofemoral instability population.

Despite the comprehensive exploration of the sub-scales and cross-sectional data, the study has some limitations. First, while the most substantial improvement occurred within the first six months post-surgery, the temporal resolution of this recovery was limited by the discrete data-collection intervals. Consequently, the precise trajectory of early improvements and changes beyond 24 months remains unclear. Additionally, while this study confirms significant improvement in BPII scores, the underlying mechanisms and individual variability in recovery were not explored. Furthermore, missing data across intervals were unavoidable due to the nature of extracting from an ongoing clinical database, which may introduce potential bias. Finally, the use of a single-centre geographical cohort may limit the external validity and generalizability of these findings to broader patient populations. Future research should integrate measures of psychological readiness, objective return-to-sport timing, and extended follow-up (> 24 months) within prospective longitudinal designs to provide a more comprehensive understanding of the factors contributing to long-term patient outcomes.

In conclusion, the findings of this study support the BPII 2.0 as a reliable, valid, and responsive disease-specific patient-reported outcome measure for individuals with patellofemoral instability. Structural validity analyses confirmed the multidimensional structure, supporting the use of both total and sub-scale scores to assess different domains of recovery. The absence of floor and ceiling effects, combined with strong responsiveness over a two-year postoperative period, indicates that the BPII 2.0 can effectively capture meaningful changes throughout recovery following MPFL reconstruction. For clinicians treating patellofemoral instability, the BPII 2.0 provides a practical tool to monitor patient-reported outcomes and better understand the physical, functional, and psychosocial factors influencing recovery. These findings support the BPII 2.0 as a clinically relevant, disease-specific outcome measure for evaluating recovery and guiding research in patients with patellofemoral instability.

**Take home message**

- The Banff Patellofemoral Instability Instrument 2.0 identifies domain-specific recovery deficits.

- Recreation and psychological recovery lag behind physical recovery.

- These insights guide targeted interventions and return-to-sport decision-making.

## Data Availability

The data that support the findings for this study are available to other researchers from the corresponding author upon reasonable request.
